# Accurate detection of cholangiocarcinoma in primary sclerosing cholangitis using DNA methylation biomarkers in bile and plasma

**DOI:** 10.1016/j.jhepr.2026.101876

**Published:** 2026-04-29

**Authors:** Hege Marie Vedeld, Sigurd Breder, Heidi Pharo, Hans Petter Brodal, Evy Marie Adolfsen, Sara Brandt-Winge, Erik von Seth, Marit Mæhle Grimsrud, Sheraz Yaqub, Tom H. Karlsen, Krzyztof Grzyb, Vemund Paulsen, Martti A. Färkkilä, Annika Bergquist, Lars Aabakken, Kirsten M. Boberg, Trine Folseraas, Guro E. Lind

**Affiliations:** 1 Department of Molecular Oncology, Institute for Cancer Research, Oslo University Hospital - the Norwegian Radium Hospital, Oslo, Norway; 2 Norwegian PSC Research Center, Department of Transplantation Medicine, Oslo University Hospital, Oslo, Norway; 3 Institute of Clinical Medicine, University of Oslo, Oslo, Norway; 4 Department of Medicine Huddinge, Unit of Gastroenterology and Nutrition (GUT), Karolinska Institutet, Karolinska University Hospital, Stockholm, Sweden; 5 Department of Oncology, Akershus University Hospital, Lørenskog, Norway; 6 Section of Hepato-Pancreato-Biliary (HPB) Surgery, Department of Gastrointestinal and Pediatric Surgery, Oslo University Hospital, Oslo, Norway; 7 Section of Gastroenterology, Department of Transplantation Medicine, Division of Surgery, Inflammatory Medicine and Transplantation, Oslo University Hospital - Rikshospitalet, Oslo, Norway; 8 Department of Pathology, Oslo University Hospital, Rikshospitalet, Oslo, Norway; 9 Helsinki University and Department of Medicine, Division of Gastroenterology, Helsinki University Hospital, Helsinki, Finland; 10 Department of Biosciences, The Faculty of Mathematics and Natural Sciences, University of Oslo, Oslo, Norway

**Keywords:** Bile, Biliary tract cancer, Biomarker, Cholangiocarcinoma, ctDNA, ddPCR, Epigenetics, Plasma, Primary sclerosing cholangitis, Surveillance

## Abstract

**Background & Aims:**

Current methods for cholangiocarcinoma (CCA) detection in primary sclerosing cholangitis (PSC) lack accuracy, often leading to late, non-curative diagnoses. In this study, we aim at identifying DNA methylation biomarkers in liquid biopsies for earlier and more accurate CCA detection.

**Methods:**

Reduced representation bisulfite sequencing was used to analyze tissue samples from individuals with CCA (n = 10) and PSC (n = 9), yielding 12 candidate DNA methylation biomarkers. After droplet digital PCR assay development, quality control, and verification in tissue, we selected four biomarkers for evaluation in bile (n = 272) and plasma (n = 128) samples from individuals with PSC and/or CCA collected across three different hospitals.

**Results:**

Four biomarkers demonstrated perfect discrimination (AUC = 1.00) between PSC-associated CCA (PSC-CCA) and PSC alone in tissue samples. In bile, all four biomarkers achieved an AUC >0.9 for distinguishing PSC-CCA (n = 20) from PSC alone (n = 207). A two-biomarker panel (5-42952178 and *PRKCB)* achieved 95% sensitivity and 90% specificity; the addition of *KCNA6* and *ZFP82* increased the specificity to 97% while maintaining the sensitivity at 90%. Notably, the two-biomarker bile panel successfully detected all eight CCA cases (100% sensitivity) in samples collected 3–12 months before diagnosis using conventional methods. Performance was lower in plasma, where an alternative two-biomarker panel (*KCNA6* and *PRKCB*) achieved 70% sensitivity and 90% specificity for distinguishing PSC-CCA (n = 17) from PSC alone (n = 101).

**Conclusions:**

Four DNA methylation biomarkers accurately discriminate CCA from PSC in bile, with lower performance in plasma. Their ability to detect CCA months before clinical diagnosis supports further evaluation in prospective studies to enable earlier, potentially curative intervention in PSC.

**Impact and implications:**

In this study, we demonstrate that liquid biopsy-based DNA methylation biomarkers offer high sensitivity and specificity for early detection of CCA in individuals with PSC, directly addressing a critical unmet need. Early and accurate detection may enable timely surgical intervention, with the potential to significantly improve survival in this high-risk group. Both bile and plasma samples are accessible via routine clinical care, and implementation of such biomarkers would not introduce additional procedural risk. If validated in prospective studies, these biomarkers could transform standard CCA surveillance in PSC by providing a minimally invasive, reliable tool for longitudinal monitoring and earlier detection and intervention.

## Highlights

• DNA methylation biomarkers in liquid biopsies allow reliable CCA detection in PSC.
• A two-biomarker bile panel shows 95% sensitivity and 90% specificity.
• The biomarker panel detects CCA up to 1 year earlier than current standard methods.
• A two-biomarker plasma panel shows 70% sensitivity and 90% specificity.
• Potential to improve surveillance and earlier, curative interventions in PSC.

## Graphical abstract

**Figure:**
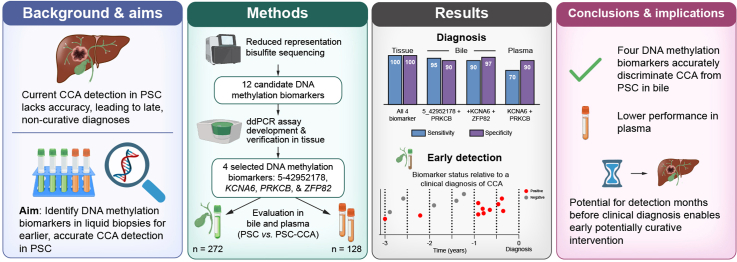

## Introduction

Primary sclerosing cholangitis (PSC) is a chronic, progressive cholestatic liver disease characterized by inflammation, fibrosis, and multifocal strictures of the bile ducts, leading to end-stage liver disease in the majority of cases.^1^ PSC has a fluctuating disease course, a highly variable disease severity, and no effective disease-modifying therapies.^2^ Individuals with PSC have a markedly increased risk of cancer, particularly cholangiocarcinoma (CCA), which is among the leading causes of death in this patient group.[3], [4], [5], [6] Early detection of CCA in PSC can enable curative treatment by liver resection or transplantation and considerably improve survival.^7^^,^^8^ However, current CCA screening strategies are restricted by their low diagnostic accuracy.^9^

CCA is a malignancy that emerges in the biliary tree; it is classified according to the anatomical origin as intrahepatic, perihilar, or distal CCA.^10^ Most cases are sporadic; however, in the Western world, PSC is the most common established risk factor for CCA.^11^^,^^12^ Although CCA is rare in the general population,^11^ individuals with PSC have a markedly increased lifetime risk (15–20%),^13^^,^^14^ with yearly incidence rates of 0.5–1.5%.^14^^,^^15^ In PSC, CCA may develop through a dysplasia-carcinoma sequence,^16^ suggesting that surveillance for early CCA detection is feasible.^17^ However, distinguishing between benign and malignant bile duct strictures in PSC remains challenging,^18^ warranting the use of multiple modalities, including radiological imaging, serum tumor marker carbohydrate antigen 19-9 (CA 19-9), endoscopic retrograde cholangiopancreatography (ERCP), and/or cholangioscopy with tissue sampling.^19^ Despite these approaches, diagnostic accuracy is suboptimal,^13^ underscoring the urgent need for reliable biomarkers for early detection of CCA.

Epigenetic alterations, particularly aberrant DNA methylation, are common in both benign and malignant tumors, and show promise as early detection biomarkers.^20^ DNA methylation biomarkers have been detected in plasma, serum, and various other body fluids from individuals with gastrointestinal (GI) cancer,^20^^,^^21^ including CCA,^21^^,^^22^ supporting the potential for liquid biopsy-based early detection of CCA.

In this study, we aim at identifying novel DNA methylation biomarkers that can accurately differentiate CCA from PSC by analyzing bile and plasma samples. Candidate biomarkers were discovered through methylome sequencing of tissue samples from individuals with CCA and PSC; they were subsequently validated by droplet digital PCR (ddPCR) in bile and plasma from individuals with CCA and/or PSC, and with non-malignant liver disease other than PSC.

## Materials and methods

### Patient samples: tissue, bile, and plasma

In this study, we included samples collected from individuals diagnosed with PSC alone (without any malignancy), PSC with concomitant CCA (PSC-CCA), sporadic CCA (no concomitant PSC), and non-malignant liver disease other than PSC (disease controls), including hereditary liver disease, non-alcoholic fatty liver disease, biliary stone disease, and autoimmune liver disorders. The samples were sourced from Oslo University Hospital (OUS), Rikshospitalet (Oslo, Norway; tissue, bile, and plasma samples), Karolinska University Hospital (Stockholm, Sweden; bile samples), and Helsinki University Hospital (Helsinki, Finland; bile samples) between 2008 and 2019. As previously described,^23^ the diagnosis of PSC was based on typical findings on magnetic resonance cholangiography and/or ERCP as defined by established criteria.^5^^,^^24^ The diagnosis of CCA was confirmed by histopathological analysis of tissue samples or through a combination of clinical, biochemical, radiological, and cytological assessments.^5^^,^^25^ Available biliary brush cytology results were classified as follows: 0, acellular; 1, normal; 2, atypia; 3, suspicious of carcinoma; and 4, diagnostic of carcinoma. Individuals with CCA who were receiving chemotherapy at the time of sampling were excluded from the study.

#### Tissue: PSC, PSC-CCA, and sporadic CCA

We included 19 fresh frozen tissue samples from individuals with CCA (n = 10, three PSC-CCA and seven CCA) and PSC (n = 9). Samples were obtained from individuals undergoing surgery at OUS between 2008 and 2012; they were snap-frozen immediately after surgery and stored at -80°C. Samples were processed and evaluated as previously described.^26^ See Table S1 for detailed clinical information.

#### Bile samples: PSC, PSC to CCA, PSC-CCA, sporadic CCA, and disease controls

We included a total of 272 bile samples, obtained at OUS (n = 218), Karolinska University Hospital (n = 12), and Helsinki University Hospital (n = 42) between 2008 and 2019. Of 272 samples, 41 were from individuals who had or developed CCA within 36 months after bile sampling (35 of these had underlying PSC), 207 were from individuals with PSC alone, and 24 were from disease controls. Bile was collected during ERCP (if possible before injecting contrast medium), at liver transplantation, or on one occasion during liver resection. All samples were stored at -80°C or -20°C (Karolinska University Hospital). See Tables S2 and S3 for detailed clinical information.

For analysis, samples were divided into two main groups: individuals with PSC (group 1, n = 242) and individuals without PSC (group 2, n = 30) (Fig. S1). The main focus of this article was on group 1, including (1) individuals diagnosed with concomitant PSC and CCA (clinical sampling performed <3 months before the diagnosis of CCA; PSC-CCA, n = 20), (2) individuals with PSC who were diagnosed with CCA 3–36 months after clinical sampling (PSC to CCA, n = 15), and (3) individuals with PSC alone (no diagnosis of CCA >36 months after clinical sampling, n = 207). Table 1 presents demographic and clinical characteristics of individuals with PSC (n = 242). Table 2 presents histopathological data for individuals with PSC and CCA (n = 35, including both PSC-CCA and PSC to CCA). Group 2 included individuals with sporadic CCA (no underlying PSC, n = 6) and disease controls (n = 24; Tables S2 and S3).

**Table 1:** Demographic and clinical characteristics in 242 patients with primary sclerosing cholangitis with available bile samples, separated by groups.

	All patients with PSC (N = 242)	PSC with CCA (n = 35)∗	PSC alone (n = 207)
Sex			
Female	58 (24.0)	9 (25.7)	49 (23.7)
Male	184 (76.0)	26 (74.3)	158 (76.3)
Age at PSC diagnosis (yr)	33.5 (25.4–46.7)	48.8 (37.3–57.2)	31.4 (23.3–45.2)
Age at CCA diagnosis (yr)	NA	53.2 (37.6–57.9)	NA
Inflammatory bowel disease (IBD)			
IBD	173 (71.5)	25 (71.4)	148 (71.5)
No IBD	59 (24.4)	9 (25.7)	50 (24.2)
Unknown	10 (4.1)	1 (2.9)	9 (4.3)
Stage of liver fibrosis at the time of sampling†			
Cirrhosis	44 (20.6)	5 (20.8)	39 (20.5)
No cirrhosis	170 (79.4)	19 (79.2)	151 (79.5)
Acute cholangitis at the time of sampling‡			
Yes	3 (1.3)	2 (5.7)	1 (0.5)
No	237 (98.7)	33 (94.3)	204 (99.5)
CA 19-9 (kU/L) before sampling§	21 (10–42)	52 (17–599)	19 (10–36)
Dead¶	46 (19.0)	29 (82.9)	17 (8.2)

Data are presented as n (%) or median (IQR). All percentages are presented as valid percent.

CCA, cholangiocarcinoma; NA, not available; PSC, primary sclerosing cholangitis.

∗ Includes patients with PSC-CCA (n = 20) and PSC to CCA (n = 15).

† Missing information in 28/242: n = 11 in group PSC-CCA, n = 17 in group PSC alone.

‡ Missing information in 2/242: n = 2 in group PSC alone.

§ Missing information in 11/242: n = 3 in group PSC-CCA, n = 8 in group PSC alone.

¶ Deceased by 15 November 2023.

**Table 2:** Histopathological data for patients with PSC and CCA∗ and bile samples.

	n (%)
Tumor location of CCA	
Intrahepatic	12 (34.3)
Perihilar	15 (42.9)
Distal	6 (17.1)
Intrahepatic/perihilar	1 (2.9)
Unclassifiable subtype	1 (2.9)
AJCC (8th edition)	
AJCC 0	1 (2.9)
AJCC I	1 (2.9)
AJCC II	5 (14.3)
AJCC III	15 (42.9)
AJCC IV	8 (22.9)
NA	5 (14.3)
T - Primary tumor	
Tis (carcinoma *in situ*)	1 (2.9)
T1	4 (11.4)
T2	12 (34.3)
T3	4 (11.4)
T4	6 (17.1)
TX	8 (22.9)
N - Regional lymph nodes	
N0 (no regional lymph node metastasis)	5 (14.3)
N1 (one to three positive lymph nodes)	19 (54.3)
NX (regional lymph nodes cannot be assessed)	11 (31.4)
M - Distant metastasis	
M0 (no distant metastasis)	27 (77.1)
M1 (distant metastasis)	8 (22.9)
Resection margin	
R0	9 (25.7)
R1	5 (14.3)
NA	21 (60.0)
Perineural growth	
Yes	11 (31.4)
No	2 (5.7)
NA	22 (62.9)
Vascular encasement	
Yes	6 (17.1)
No	9 (25.7)
NA	20 (57.1)
Grade (G) of differentiation	
Low (G3)	4 (11.4)
Medium (G2)	10 (28.6)
High (G1)	1 (2.9)
NA	20 (57.1)
Growth pattern	
Mass-forming	9 (25.7)
Periductal-infiltrating	4 (11.4)
Intraductal-growing	1 (2.9)
Mass-forming and periductal-infiltrating	2 (5.7)
NA	19 (54.3)

The stage is from the time of diagnosis.

AJCC 0–IV, American Joint Committee on Cancer classification stages 0–IV; CCA, cholangiocarcinoma; NA, not available; PSC, primary sclerosing cholangitis.

∗ Includes patients with PSC-CCA (n = 20) and PSC to CCA (n = 15).

The bile samples included in this study largely overlapped with those from a previous study.^23^

#### Plasma samples: PSC, PSC to CCA, PSC-CCA, and sporadic CCA

Plasma samples were available for a subset of patients (n = 128/218, 58.7%) collected at OUS. Of these, 20 patients had CCA at the time of plasma sampling (17 PSC-CCA, three sporadic CCA), seven developed CCA 3–12 months after sampling (PSC to CCA), and 101 patients had PSC alone (Fig. S1). Plasma was separated from whole blood using a two-step centrifugation protocol. The first centrifugation step was performed at 1,500*g* for 15 min and the second at 15,000*g* for 15 min. Plasma samples were stored at -80°C until DNA extraction. See Table S4 for detailed clinical information, Table 3 for demographic and clinical characteristics of individuals with PSC (n = 125), and Table 4 for histopathological data of individuals with PSC and CCA (n = 24, including both PSC-CCA and PSC to CCA).

**Table 3:** Demographic and clinical characteristics in 125 patients with primary sclerosing cholangitis with available plasma samples, separated by groups.

	All patients with PSC (n = 125)	PSC with CCA (n = 24)∗	PSC alone (n = 101) (%)
Sex			
Female	26 (20.8)	6 (25.0)	20 (19.8)
Male	99 (79.2)	18 (75.0)	81 (80.2)
Age at PSC diagnosis (years)	34.1 (27.1–49.3)	52.5 (38.0–59.0)	31.0 (26.2–44.8)
Age at CCA diagnosis (years)	NA	55.5 (40.2–65.4)	NA
Inflammatory bowel disease (IBD)			
IBD	87 (69.6)	18 (75.0)	69 (68.3)
No IBD	32 (25.6)	5 (20.8)	27 (26.7)
Unknown	6 (4.8)	1 (4.2)	5 (5.0)
Stage of liver fibrosis at the time of sampling†			
Cirrhosis	20 (18.5)	2 (10.0)	18 (20.4)
No cirrhosis	88 (81.5)	18 (90.0)	70 (79.6)
Acute cholangitis at the time of sampling‡			
Yes	6 (4.9)	2 (8.3)	4 (4.0)
No	117 (95.1)	22 (91.7)	95 (96.0)
CA 19-9 (kU/L) before sampling§	28 (15–60)	144 (22–967)	23 (15–45)
Dead¶	25 (20.0)	18 (75.0)	7 (6.9)

Data are presented as n (%) or median (IQR). All percentages are presented as valid percent.

CCA, cholangiocarcinoma; NA, not available; PSC, primary sclerosing cholangitis.

∗ Includes patients with PSC-CCA (n = 17) and PSC to CCA (n = 7).

† Missing information in17/125: n = 4 in group PSC with CCA, n = 13 in group PSC alone.

‡ Missing information in two of 125: n = 2 in group PSC.

§ Missing information in three of 125: n = 3 in group PSC.

¶ Deceased by 15 November 2023.

**Table 4:** Histopathological data for patients with PSC and CCA (n = 24∗) and plasma samples.

n = 24	n (%)
Tumor location of CCA	
Intrahepatic	4 (16.7)
Perihilar	14 (58.3)
Distal	6 (25.0)
AJCC (8th edition)	
AJCC 0	0
AJCC I	1 (4.2)
AJCC II	6 (25.0)
AJCC III	10 (41.7)
AJCC IV	5 (20.8)
NA	2 (8.3)
T - Primary tumor	
Tis (carcinoma *in situ*)	0
T1	2 (8.3)
T2	9 (37.5)
T3	6 (25.0)
T4	2 (8.3)
TX	5 (20.8)
N - Regional lymph nodes	
N0 (no regional lymph node metastasis)	4 (16.7)
N1 (one to three positive lymph nodes)	13 (54.2)
NX (regional lymph nodes cannot be assessed)	7 (29.2)
M - Distant metastasis	
M0 (no distant metastasis)	19 (79.2)
M1 (distant metastasis)	5 (20.8)
Resection margin	
R0	6 (25.0)
R1	4 (16.7)
NA	14 (58.3)
Perineural growth	
Yes	11 (45.8)
No	0
NA	13 (54.2)
Vascular encasement	
Yes	4 (16.7)
No	8 (33.3)
NA	12 (50.0)
Grade (G) of differentiation	
Low (G3)	2 (8.3)
Medium (G2)	11 (45.8)
High (G1)	1 (4.2)
NA	10 (41.7)
Growth pattern	
Mass-forming	5 (20.8)
Periductal-infiltrating	2 (8.3)
Intraductal-growing	0
Mass-forming and periductal-infiltrating	0
NA	17 (70.8)

The stage is from the time of diagnosis.

AJCC 0–IV, American Joint Committee on Cancer classification stages 0–IV; CCA, cholangiocarcinoma; NA, not available; PSC, primary sclerosing cholangitis.

∗ Includes patients with PSC-CCA (n = 17) and PSC to CCA (n = 7).

Tissue, bile, and plasma samples were derived from the same patient cohorts but not necessarily collected at the same time point.

### DNA isolation

We extracted DNA from ∼25 mg of fresh frozen tissue using the AllPrep® DNA/RNA Kit (Qiagen, Hilden, Germany) following the manufacturer’s recommendations. For bile, DNA was extracted from 100 to 200 μl of bile using a standard phenol-chloroform procedure. Cell-free DNA (cfDNA) was extracted from 2 ml plasma using the QIAamp® Circulating Nucleic Acid Kit (Qiagen) according to the manufacturer’s protocol. For further details, see supplementary materials and methods – DNA isolation.

### RRBS: identification of biomarker candidates

We performed reduced representation bisulfite sequencing (RRBS) of the 19 tissue samples, including 10 from patients with CCA and nine from patients with PSC, at the Genomics Core Facility, OUS (Oslo, Norway), using the NuGEN Ovation® RRBS Methyl-Seq kit (NuGEN Technologies, San Carlos, CA, USA). Each sample was sequenced in multiple runs to obtain sufficient data for downstream analysis. Details of preprocessing are provided in Supplementary Material and methods – RRBS. After processing the RRBS raw data, the resulting Binary Alignment Map (BAM) files contained an average of 65 million mapped reads, with a mean of 3.8 million CpG sites covered at a depth of at least 10 × per sample. Using a MethylSeqDesign framework,^27^ simulated RRBS data indicated that a sample size of nine per group provides at least 90% power at a 5% significance level, even at low sequencing depths (Fig. S2).

#### Differentially methylated regions

To identify differentially methylated regions (DMRs) between samples from patients with PSC and CCA, we used methylKit^28^ and DMRfinder^29^ tools with default settings. For methylKit, DMRs were defined by a sliding window approach as previously described.^30^ Briefly, significantly differentially methylated windows (FDR‑adjusted *p* <0.05) with at least 25% more methylation in the CCA group were retained and considered for further biomarker candidate selection. For DMRfinder, DMRs were defined as having three or more CpGs with ≤100 bp distance between CpGs, and the total length of the region ≤500 bp. Regions with five or more CpGs, spanning at least 50 bp, and showing at least 10% more methylation in the CCA group were retained for further biomarker candidate selection. Both methods were set up to retain only hypermethylated regions in CCA.

We hypothesized that biomarkers with no or low methylation in the PSC tissue would remain unmethylated or below the detection limit in liquid biopsies such that a positive signal in plasma or bile would reliably indicate tumor-derived methylation. To test this, we applied a bioinformatic pipeline designed to prioritize regions with minimal methylation in PSC but detectable methylation in CCA. In addition, we used a second strategy to expand the biomarker list, aiming to identify candidate regions with maximal methylation differences between CCA and PSC. For further details, see Supplementary Materials and methods – Selection of candidate biomarkers from RRBS data.

### Bisulfite conversion

DNA from tissue and bile was subjected to bisulfite conversion using the EpiTect® Bisulfite Kit (Qiagen) with an input of 1,300 ng and 500 ng, respectively. Furthermore, cfDNA from plasma was subjected to bisulfite conversion using the EpiTect® Plus Bisulfite Kit (Qiagen) with inputs of 5 to 370 ng. The manufacturers’ protocols were followed for both kits.^31^ Bisulfite-converted DNA was automatically purified using a QIAcube Connect (Qiagen) and eluted in 40 μl (tissue and bile) or 10–16 μl (plasma; cfDNA) elution buffer. Bisulfite-converted samples were stored at -20°C.

### Droplet digital PCR

The QX200™ Droplet Digital™ PCR System (Bio-Rad, Hercules, CA, USA) was used to analyze the biomarkers in tissue and bile with an input of 32.5 ng and 37.5 ng of bisulfite-converted DNA, respectively. Each biomarker was analyzed in duplex with the 4Plex internal control.^32^ The ddPCR reaction consisted of 1 × ddPCR™ Supermix for Probes (Bio-Rad), 818 nM of each primer, 182 nM of each probe, and 3 μl of bisulfite-converted DNA template in a final volume of 22 μl. For cfDNA, the QX600™ Droplet Digital™ PCR System (Bio-Rad) was used for multiplex analysis of the four best-performing DNA methylation biomarkers from tissue, including the 4Plex internal control. The input ranged 4–25 ng of bisulfite-converted cfDNA. The ddPCR reaction consisted of 1 × ddPCR™ Multiplex Supermix, 818 nM of each primer, 182 nM of each probe, and 1.1–8.5 μl bisulfite-converted DNA template in a final volume of 22 μl. For further details on droplet generation and analyses, see Supplementary Materials and methods – Droplet digital PCR.

Analyses were performed according to the minimum information for publication of quantitative digital PCR experiments (dMIQE) guidelines^33^ (Table S5) and the STAndards for the Reporting of Diagnostic accuracy studies (STARD) checklist^34^ (Table S6).

#### ddPCR assay design and technical validation of ddPCR assays

For optimal design of ddPCR assays, candidates/regions from RRBS were visually inspected in the Integrative Genomics Viewer^35^ using bedGraph files of the extracted methylation calls from both methylKit and DMRfinder. In total, 12 ddPCR assays were designed. Primer and probe sequences are listed in Table S7. All assays were quality controlled on the ddPCR platform, including methylation-positive and methylation-negative controls (universal methylated human DNA standard and normal blood, respectively), and non-template controls. Eight assays passed the quality control (no or very little methylation in normal blood) and were further validated in the same 10 CCA and nine PSC tissue samples previously analyzed by RRBS.

### Statistical methods for ROC curve analyses and sample size estimation

We performed power calculations for receiver operating characteristic (ROC) analyses using the R package pROC (R Foundation for Statistical Computing, Vienna, Austria).^36^ Based on the fixed sample sizes (available material), we calculated the minimum detectable AUC at α = 0.05 and 80% power. With 20 cases and 207 controls (PSC-associated CCA: bile), the resulting minimum detectable AUC was 0.685. For PSC-associated CCA in plasma (17 cases and 101 controls) it was 0.706, and 0.845 for sporadic CCA in bile (six cases and 24 controls). We performed power calculations for observed AUCs at α = 0.05 (Table S8). For each biomarker, ROC curve analysis and AUC estimates were conducted with bootstrap-derived 95% CIs using 10,000 resamples. Two-sided bootstrap *p* values were calculated for the null hypothesis AUC = 0.5, and *p* <0.05 was considered statistically significant. All analyses were performed in R (version 4.4.2; R Foundation for Statistical Computing) using the pROC and boot packages. To ensure reproducibility, a fixed random seed was set before resampling. ROC curves and AUC estimates for tissue samples were generated with GraphPad Prism version 10.4.2 (GraphPad Software, San Diego, CA, USA).

### Biomarker thresholding and selection of biomarker panels

For each biomarker, a threshold was selected from the ROC curve to maximize sensitivity while maintaining a specificity of at least 90%. Samples with missing values were excluded. Biomarkers were then combined into panels by evaluating every possible combination of the four binary biomarkers, applying all possible k-of-n decision thresholds for each combination (*i.e.* the rule is positive if at least k of the n markers in the subset are positive; *k* = 1, …, *n*). Sensitivity and specificity were calculated for all biomarker combinations and thresholds. Furthermore, 95% CIs were estimated using stratified bootstrap resampling (10,000 iterations) with replacement separately within cases and controls. The best-performing panel focusing on sensitivity was defined as the combination yielding the highest sensitivity among those with a specificity of at least 0.90. Additionally, we reported on the best-performing panel focusing on specificity, defined as the combination yielding the highest specificity among those with a sensitivity of at least 0.90.

### Ethics

In cases of long-archived samples, where obtaining written informed consent was not possible, the local ethics committee granted an exception from informed consent to allow use of the samples. All of the remaining participants provided written informed consent. The study protocols were approved by ethics committees of all the recruiting centers, as well as the Regional Committee for Medical and Health Research Ethics South-Eastern Norway (REK id 28290), the Swedish Ethical Review Authority (2013/2084-31/2 with amendment 2016/1023, 2018/1786-32), and the Helsinki University Hospital Ethical Committee IV (HUS/1566/2020).

## Results

**Fig. 1:**
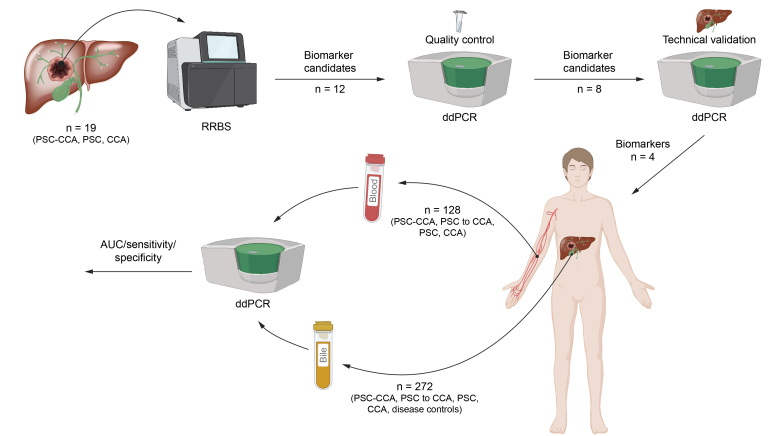
**Overall strategy used to identify biomarker candidates, and evaluation of the biomarkers in bile and plasma samples from individuals with PSC-CCA, PSC to CCA, PSC alone, sporadic CCA, and disease controls**. CCA, cholangiocarcinoma; ddPCR, droplet digital PCR; PSC, primary sclerosing cholangitis; disease controls, non-malignant liver diseases other than PSC; ROC, receiver operating characteristics; RRBS, reduced representation bisulfite sequencing.

We performed RRBS analyses of the tissue samples to identify candidate DNA methylation biomarkers for early detection of CCA in individuals with PSC. These candidates were validated for accuracy by ddPCR analysis of bile and plasma samples. Fig. 1 illustrates the stepwise strategy for biomarker discovery and validation.

### Performance of biomarker candidates

To assess the discriminatory power of the eight selected DNA methylation biomarkers, we measured their normalized methylation levels in CCA and PSC tissue samples by ddPCR, using the same tissue samples that had been analyzed by RRBS. All assays demonstrated significantly higher methylation levels in CCA than in PSC (*p* <0.005; Fig. S3). Correspondingly, the AUCs for differentiating CCA from PSC ranged from 0.90 to 1.00 (Fig. S4). Four assays *PRKCB* (protein kinase C beta), *KCNA6* (potassium voltage-gated channel subfamily A member 6), *ZFP82* (zinc finger protein 82), and 5-42952178 (genomic location) displayed perfect discrimination (AUC = 1.00) and were selected for further validation in bile and plasma samples. Two of the biomarkers (*PRKCB* and *ZFP82*) were completely unmethylated in PSC tissue samples.

Among the 19 individuals included in the tissue analysis, 12 also had corresponding bile samples available (four CCA and eight PSC). We observed a strong correlation between methylation levels in bile and the corresponding tissue sample (Fig. S5). Of note, bile and tissue were rarely collected at the same time point, but most paired samples were obtained <1 year apart.

### Validation in bile samples: Potential for more accurate diagnostics

The four assays that achieved perfect discrimination (AUC = 1.00) in tissue samples were further evaluated in 242 bile samples: 20 from patients with PSC-CCA, 15 from patients who developed CCA 3–36 months after bile sampling (PSC to CCA), and 207 from individuals with PSC alone. Patient characteristics are summarized in Table 1, Table 2 and Table S2.

**Fig. 2:**
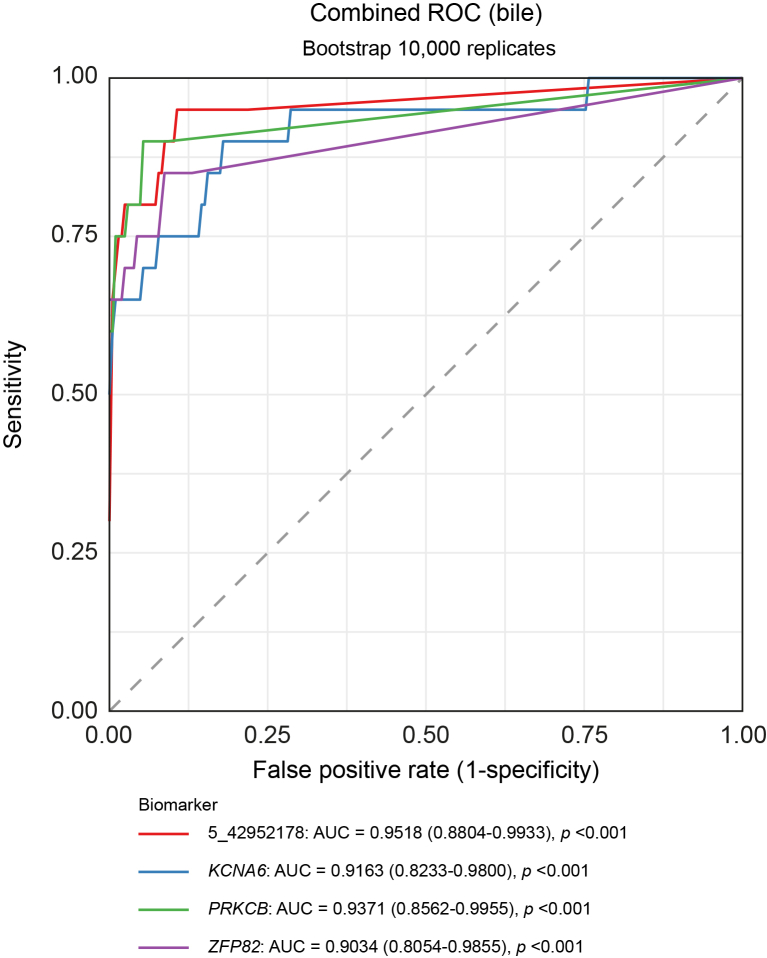
Combined ROC curves for biomarkers analyzed in bile from individuals with PSC-CCA (n = 20) and PSC-control (n = 207). ROC curves and AUCs were computed in R (pROC package). The 95% CIs were estimated by bootstrapping (B = 10,000; boot package). Two-sided bootstrap *p* values were calculated for the null hypothesis AUC = 0.5, and *p* <0.05 was considered statistically significant. CCA, cholangiocarcinoma; PSC, primary sclerosing cholangitis; ROC, receiver operating characteristics.

ROC curve analyses comparing bile samples from PSC-CCA with those from PSC alone demonstrated a strong discriminatory performance for all four biomarkers, with AUCs of 0.952 (5-42,952,178), 0.916 (*KCNA6*), 0.937 (*PRKCB*), and 0.903 (*ZFP82*) (Fig. 2). Using ROC curve-derived thresholds, the sensitivities and specificities were 90% and 91% for 5-42,952,178, 75% and 92% for *KCNA6*, 90% and 95% for *PRKCB*, and 85% and 91% for *ZFP82,* respectively (Table S9). Individual normalized methylation levels by diagnostic group (PSC-CCA, PSC to CCA, PSC-control) are shown in Fig. S6.

We then evaluated biomarker panels for optimal diagnostic performance. Prioritizing sensitivity, the combination of 5-42,952,178 and *PRKCB* achieved a sensitivity of 95% and specificity of 90% when at least one biomarker was methylated (Table S10). Prioritizing specificity, the four-biomarker panel reached a specificity of 97% and sensitivity of 90% when at least three biomarkers were methylated.

Notably, seven individuals with PSC alone developed CCA >3 years after bile collection. Among these patients, the two-biomarker panel was positive in two (29%) and the four-biomarker panel was positive in one (14%).

### Performance in bile samples: Potential for early detection

**Fig. 3:**
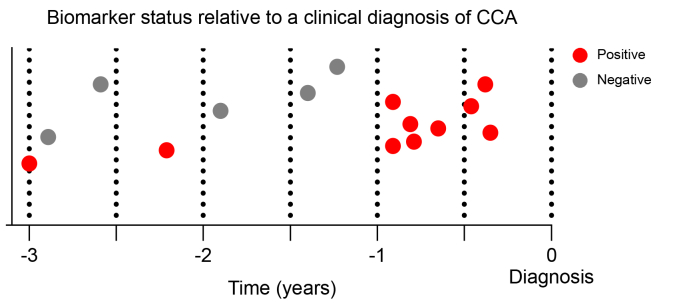
Biomarker status relative to CCA diagnosis by conventional methods. A red circle indicates a sample positive for the two-biomarker panel (5-42952178 and *PRKCB)*, whereas a gray circle denotes a sample negative for the biomarker panel. The y-axis does not correspond to a quantitative variable; data points are jittered vertically to reduce overlap and improve visualization of the distribution of methylation values across samples. Time 0 indicates the point of CCA diagnosis by conventional methods. CCA, cholangiocarcinoma.

To evaluate whether the biomarker panels could detect CCA earlier than current methods, we analyzed the PSC to CCA samples (n = 15), in which bile samples were collected 3–36 months before diagnosis by conventional methods. The thresholds applied for biomarker positivity were identical to those previously defined for the PSC-CCA samples. Strikingly, the two-biomarker panel (5-42,952,178 and *PRKCB*) detected CCA with 100% sensitivity in cases where bile was obtained 3–12 months before diagnosis (n = 8; Fig. 3) The four-biomarker panel was positive in seven of these eight cases (87.5% sensitivity; Table S11). For samples collected more than 12 months before diagnosis (n = 7), the sensitivity for both panels declined to 29% (n = 2/7; Fig. 3).

Notably, the analyzed patient cohort included diverse tumor growth patterns, anatomical locations, cancer stages at diagnosis, and tumor sizes (Tables 2 and S2). This indicates that the presence of positive methylation biomarkers is independent of pathological features that influence the clinical presentations of CCA. For further details on the associations between the two-biomarker panel methylation levels and clinical or molecular features in the 35 patients with PSC-CCA and PSC to CCA, see Table S12.

### Performance of the biomarker panels *vs*. brush cytology

ERCP with biliary brush cytology remains a valuable tool for CCA detection in PSC,^37^ and was performed in 194 of 242 (80%) patients with PSC included in this study (Table S2). Among these, 25 samples were classified as “suspicious of carcinoma”: 10 samples from individuals with CCA (n = 8 PSC-CCA, n = 2 PSC to CCA), and 15 from those with PSC alone. The two- and four-biomarker panels were positive in all CCA samples classified as “suspicious of carcinoma”, whereas only three and two of the 15 PSC alone samples were positive, respectively.

Brush cytology was scored as “atypia” in 32 samples, including eight CCA (n = 2 PSC-CCA, n = 6 PSC to CCA) and 24 PSC alone samples. The two- and four-biomarker panels were positive in seven and five CCA samples, respectively, and in only three PSC alone samples (Table S2).

### Performance in plasma samples

Biomarker performance was also assessed in plasma samples collected from patients with PSC-CCA (n = 17), patients who developed CCA 3–12 months after sampling (PSC to CCA, n = 7), and in 101 patients with PSC alone. All plasma samples were obtained from patients who also provided bile samples, although not necessarily at the same time point. Patient characteristics are summarized in Tables 3, 4, and S4.

**Fig. 4:**
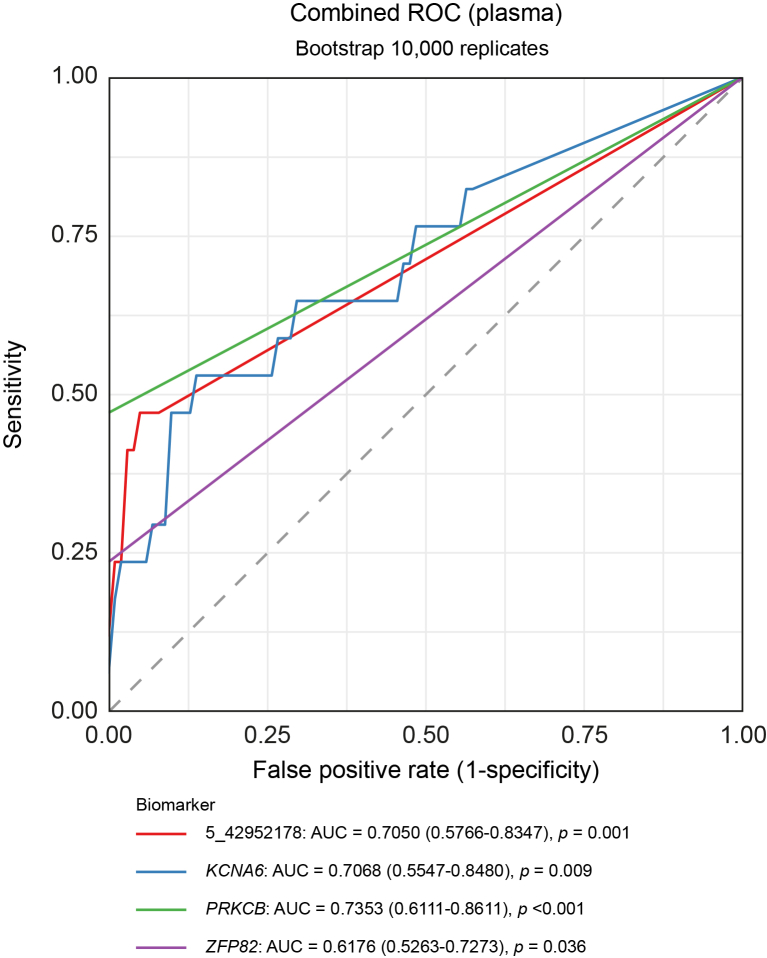
Combined ROC curves for biomarkers analyzed in plasma from individuals with PSC-CCA (n = 17) and PSC-control (n = 101). ROC curves and AUCs were computed in R (pROC package). The 95% CIs were estimated by bootstrapping (B = 10,000; boot package). Two-sided bootstrap *p* values were calculated for the null hypothesis AUC = 0.5, and *p* <0.05 was considered statistically significant. CCA, cholangiocarcinoma; PSC, primary sclerosing cholangitis; ROC, receiver operating characteristics.

ROC curve analyses comparing PSC-CCA (n = 17) and PSC alone (n = 101) samples showed that the AUCs ranged from 0.618 for *ZFP82* to 0.735 for *PRKCB* (Fig. 4). The sensitivities were 47% for 5-42952178, *KCNA6*, and *PRKCB,* and 24% for ZFP82, whereas the specificities were 95% for 5-42952178, 90% for *KCNA6*, and 100% for both *PRKCB* and *ZFP82* (Table S13).

As with bile, we evaluated combinations of biomarkers to differentiate PSC-CCA from PSC alone in plasma samples. Prioritizing sensitivity, the most optimal plasma panel was the combination of *KCNA6* and *PRKCB* (with or without *ZFP82),* achieving 71% sensitivity and 90% specificity when at least one biomarker was methylated (Table S14). Conversely, several combinations achieved a specificity of 100%, but with a sensitivity of only 47%, which did not surpass the performance of *PRKCB* alone (Table S11). Notably, the panel with the highest sensitivity in bile (5-42,952,178, *PRKCB*) achieved 53% sensitivity and 95% specificity in plasma.

When the biomarker panels were analyzed in plasma samples from the PSC to CCA group (n = 7), only one sample was positive regardless of the biomarker panel (Table S15).

### CCA and non-malignant disease controls: Bile and plasma samples

Although the main focus of this study was on individuals with PSC, we also analyzed bile samples from six patients with sporadic CCA (no underlying PSC) and 24 disease controls. Plasma samples were also available from three patients with sporadic CCA. ROC curve analyses showed that all four biomarkers could differentiate sporadic CCA from disease controls, with AUCs of 0.895 (5-42,952,178), 0.854 (*KCNA6*), 0.979 (*PRKCB*), and 0.812 (*ZFP82*) (Fig. S7). The best-performing biomarker, *PRKCB,* achieved a sensitivity of 100% and a specificity of 96% (Fig. S7 and Table S16). For plasma, two of the three sporadic CCA samples (67%) were positive for the two-biomarker panel *KCNA6* and *PRKCB,* using the thresholds established for patients with PSC (Table S4).

## Discussion

Early detection of CCA is essential for identifying malignancy at a stage where patients are still eligible for curative treatment. However, despite significant efforts, we are still lacking a non-invasive and reliable test for early CCA diagnosis in individuals with PSC. In the current study, we have identified four DNA methylation biomarkers which, when combined into panels, could differentiate PSC-associated CCA from PSC alone with high sensitivity and specificity in bile samples. Notably, the two-biomarker panel detected CCA in all bile samples collected up to 1 year before diagnosis by conventional methods. These findings suggest that the biomarker panels offer improved diagnostic accuracy for CCA compared with standard methods and, importantly, enable earlier detection. Furthermore, high sensitivity and specificity were maintained for CCA detection in plasma, suggesting that this approach facilitates less invasive, longitudinal monitoring of CCA development in individuals with PSC.

Although the biomarker panels were less reliable for detecting CCA when bile samples were collected >1 year before diagnosis, there were still more positive samples in this group than in individuals with PSC alone. Moreover, individuals with PSC who developed CCA >3 years after bile collection had higher methylation levels than those who did not develop CCA. Early DNA methylation changes are a well-established phenomenon in carcinogenesis,^38^^,^^39^ with alterations observed in preneoplastic lesions across several malignancies, including colorectal adenomas^40^ and biliary intraepithelial neoplasia.^41^ Our observations align with this, indicating that DNA hypermethylation of the current biomarkers occur early in cancer development and that individuals with positive methylation at these loci are at an increased risk of developing CCA in the future, even in the absence of a clinically detectable tumor.

Our results further indicate that the DNA methylation biomarker panels can exclude malignancy and increase diagnostic confidence in cases where brush cytology is classified as “suspicious of carcinoma.” Notably, all cancer samples in this category were positive for two-and four-biomarker panels, whereas most PSC alone samples were negative. The presence of atypical cells on cytology represents another clinical challenge for which there are no standardized management guidelines. In these cases, the biomarker panels also showed potential to distinguish CCA from PSC alone. Together, these results show the potential of the biomarker panels to improve clinical decision-making and treatment planning by better stratifying malignancy risk.

Both bile and plasma are attractive sources for biomarker discovery in CCA, but the optimal liquid biopsy source for CCA detection remains to be determined. Bile is a local source abundant in DNA, making it a valuable specimen for molecular analysis. Importantly, bile can be safely collected during diagnostic or therapeutic ERCP procedures,^8^^,^^42^ without additional procedural risk to the patients. This integration into routine care makes bile a particularly valuable source for biomarker discovery and early CCA detection in individuals with PSC. The value of bile as a biomarker source has also been highlighted by others, who have identified promising cfDNA mutations in bile that help differentiate CCA from PSC^43^ and malignant from benign biliary strictures.^44^ In our study, bile appeared to be the most reliable source for biomarker analyses compared with plasma cfDNA, considering its higher diagnostic accuracy. This has also been observed in other studies. Gou *et al.*^45^ found that bile cfDNA had significantly higher concordance in detecting somatic single-nucleotide variants/indels than plasma cfDNA (99% *vs*. 78%). Similarly, Driescher *et al.*^46^ reported that 96% of pathogenic tissue mutations could be detected in bile cfDNA, whereas plasma cfDNA showed a much lower detection rate of 32%. They further observed that only half of the bile cfDNA-detected mutations were concordantly detected in matched plasma from the same patients. In the present study, we found a strong correlation between tissue and bile DNA methylation levels in the 12 paired samples, indicating that bile DNA reliably reflects the methylation patterns in the corresponding tissue.

Using plasma for early cancer detection is attractive for cancer surveillance, considering its ease of collection, low complication risk, and high patient compliance. However, limited amounts of circulating tumor DNA in plasma may restrict sensitivity.^47^ In our study, plasma biomarkers differentiated CCA from PSC with high accuracy, but the sensitivity was lower compared with bile. Despite extracting DNA from 2 ml plasma, *vs*. 100–200 μl for bile, the DNA yield from bile samples was substantially higher (Supplementary Materials and methods – DNA isolation). To maximize the information obtained from limited plasma amounts, we developed a multiplex assay that enabled simultaneous analysis of all four biomarkers in a single reaction. However, the overall DNA quantity may still have been insufficient for optimal biomarker detection. We hypothesize that increasing both plasma volume and total DNA input could potentially enhance assay sensitivity. This is supported by previous studies demonstrating that the amount of DNA markedly influences the sensitivity of mutation assays, with higher input amounts correlating with increased sensitivity.^48^ Unfortunately, we did not have access to more plasma for further analysis in this study. As increasing the number of brushing passes during ERCP enhances the diagnostic yield for cytology,^49^ repeated blood collection may similarly enhance sensitivity. Nevertheless, the biomarkers analyzed in this study enabled earlier cancer detection in bile—a benefit not observed with plasma-based analysis.

GI tumors often share DNA methylation aberrations, and this also applies to the biomarkers identified in our study. Two biomarkers (5-42,952,178 and *PRKCB*) have overlapping probes in TCGA datasets and show hypermethylation across several GI cancers (Fig. S8), consistent with previous studies describing methylation of three of the four loci in these malignancies (see Supplementary Materials and methods – Discussion). These observations indicate that, although the biomarkers may still have clinical value, a signal detected in plasma cannot be assumed to originate specifically from CCA. In the PSC setting, however, where the main diagnostic challenge is distinguishing CCA from benign PSC-related strictures, absolute cancer type specificity is less critical. This concern is also less relevant for bile, as non-hepatobiliary malignancies are unlikely to produce markedly elevated DNA methylation in this compartment.

In our previous study,^23^ a bile-based DNA methylation panel demonstrated high accuracy for CCA detection. The search for additional biomarkers in the current study was not motivated by limitations of the original panel, but by the aim of identifying biomarkers suitable also for blood-based detection, given their relevance for non-invasive monitoring of patients with PSC. To pursue this goal, we used a bioinformatic pipeline designed to identify biomarkers with low background methylation in the PSC tissue. Although no candidates showed zero background methylation in PSC, relaxing this threshold enabled identification of strong candidates that validated in both bile and plasma. Although plasma performance was promising, bile remained the more accurate matrix in the PSC-CCA setting, offering superior sensitivity. Nevertheless, continued biomarker discovery remains relevant, as panel performance can vary across biomarker combinations and sample types. For example, although *PRKCB* is included in a proposed nine-marker panel for CCA detection,^22^ the panel showed limited accuracy for PSC-associated CCA, with an AUC of only 0.54 in plasma. Conversely, combining *PRKCB* with *KCNA6* in our study substantially improved detection, achieving 71% sensitivity and 90% specificity in plasma. These observations suggest that even modest differences in biomarker composition lead to marked differences in diagnostic accuracy.

The focus of this study was primarily on PSC-associated CCA; however, we also demonstrated highly promising results for sporadic CCA. Notably, *PRKCB* could differentiate sporadic CCA from disease controls in bile with 100% sensitivity and 96% specificity. In plasma, two of three sporadic CCA samples were positive for methylation. Further studies are needed to validate these findings; however, the current results indicate that these biomarkers could be useful for detecting CCA in individuals with liver diseases other than PSC.^11^

A limitation of this study is the modest number of CCA samples, which is a common challenge when studying rare cancer types such as CCA. Nevertheless, all four bile biomarkers demonstrated excellent discrimination for PSC-CCA, with AUCs well above the minimum detectable threshold given the available sample size. The corresponding CIs for observed AUCs were relatively narrow, supporting the robustness of these bile biomarkers for PSC-CCA detection despite the limited number of cases. In contrast, the blood-based PSC-CCA biomarkers had lower AUCs and consequently lower effective power within the available sample size. Consequently, their capacity was limited to detecting more pronounced differences between PSC and CCA. The number of sporadic CCA samples was low; therefore, these results should be interpreted with caution. This does not undermine their promising diagnostic potential, neither for plasma nor for sporadic CCA, but highlights the need for validation in larger cohorts. Despite the rarity of PSC-associated CCA, our cohort (cases and PSC controls) is larger than that in comparable studies,^22^^,^^47^ and samples were collected from three different centers, potentially reducing the risk of center-specific biases. Nevertheless, independent validation in larger and more diverse cohorts remains essential. Limited availability of plasma samples prevented assessment of whether increasing volume or DNA input could improve biomarker sensitivity, and precluded analysis of previously identified bile biomarkers^23^ in plasma. Bile and plasma were not always collected concurrently, limiting direct comparisons. Larger multi-center studies with expanded plasma sampling are needed to validate and extend these results.

## Conclusions

Routine implementation of an accurate DNA methylation-based liquid biopsy screening tool for individuals with PSC could enable early-stage CCA detection, allowing timely interventions such as resection or liver transplantation. The biomarkers identified in this study demonstrated high accuracy in bile for both precise diagnosis and earlier detection than conventional methods, with lower performance in plasma. If validated, these biomarkers could markedly improve outcomes and reduce the burden of CCA in individuals with PSC.

## Abbreviations

CA 19-9, carbohydrate antigen 19-9; CCA, cholangiocarcinoma; cfDNA, cell-free DNA; ddPCR, droplet digital PCR; DMR, differentially methylated regions; ERCP, endoscopic retrograde cholangiopancreaticography; GI, gastrointestinal; IBD, inflammatory bowel disease; *KCNA6*, potassium voltage-gated channel subfamily A member 6; OUS, Oslo University Hospital; *PRKCB*, protein kinase C beta; PSC, primary sclerosing cholangitis; ROC, receiver operating characteristics; RRBS, reduced representation bisulfite sequencing; *ZFP82*, zinc finger protein 82.

## Authors’ contributions

Study concept, design, and supervision: GEL. Data acquisition: HMV, SB, HP, HPB, EMT, SBW, EVS, MMG, SY, THK, KG, VP, MAF, AB, LA, KMB, TF. Data analyses: HMV, SB, HP, HPB, EMT, SBW. Data interpretation of data: HMV, SB, TF, GEL. Manuscript drafting: HMV. Manuscript revision and approval: SB, HP, HPB, EMT, SBW, EVS, MMG, SY, THK, KG, VP, MAF, AB, LA, KMB, TF, GEL.

## Data availability

Raw digital PCR data generated in this study have been deposited in Zenodo (see Table S5). RRBS data include individual-level human sequencing data classified as sensitive personal information under Norwegian and EU regulations. The ethical approval and informed consent obtained do not allow public deposition of raw or per-individual sequencing data.

## Declaration of generative AI and AI-assisted technologies in the writing process

During the preparation of this work, the authors used Perplexity (OpenAI) to assist in improving the clarity and precision of the manuscript wording. After using this tool/service, the authors reviewed and edited the content as needed and take full responsibility for the content of the publication.

## Financial support

This work was supported by the Norwegian Cancer Society (project number 216129) and the South-Eastern Norway Regional Health Authority (project number 2024032).

## Conflicts of interest

GEL and HP have a granted patent for PCR controls (4Plex PCR controls for normalization of targeted biomarkers), WO2019106149A2, US patent (US11555222B2). GEL, HMV, HPB, and TF have a provisional US-patent application # 63/930,346 “Compositions and methods for diagnosing and treating bile duct cancer.” The remaining authors declare no conflicts of interest. Please refer to the accompanying ICMJE disclosure forms for further details.
