# Supplementary material for: Accurate detection of cholangiocarcinoma in primary sclerosing cholangitis using DNA methylation biomarkers in bile and plasma
Source: JHEP Rep. 2026 Apr 29;8(8):101876. doi: 10.1016/j.jhepr.2026.101876 (PMC13380102; doi:10.1016/j.jhepr.2026.101876)
Supplement: Multimedia component 1 — . [file mmc1.docx]

**Supplementary Materials and methods**

**Accurate detection of cholangiocarcinoma in primary sclerosing cholangitis by DNA methylation biomarkers in bile and plasma**

Hege Marie Vedeld, Sigurd Breder, Heidi Pharo, Hans Petter Brodal, Evy Marie Thorkildsen, Sara Brandt-Winge, Erik von Seth, Marit Mæhle Grimsrud, Sheraz Yaqub, Tom H. Karlsen, Krzyztof Grzyb, Vemund Paulsen, Martti A Färkkilä, Annika Bergquist, Lars Aabakken, Kirsten M. Boberg, Trine Folseraas, Guro E. Lind

**Table of Contents**

[**Supplementary Methods** 2](#_Toc222754262)

[DNA isolation 2](#_Toc222754263)

[Reduced representation bisulfite sequencing 3](#_Toc222754264)

[Droplet digital PCR 4](#_Toc222754265)

[**Supplementary Discussion** 5](#_Toc222754266)

[**Supplementary Figures** 6](#_Toc222754267)

[Fig. S1 Flowchart. 6](#_Toc222754268)

[Fig. S2 Contour plot for genome wide power. 7](#_Toc222754269)

[Fig. S3 Tree plot of normalized DNA methylation levels in tissue. 8](#_Toc222754270)

[Fig. S4 ROC curves - tissue samples. 9](#_Toc222754271)

[Fig. S5 Correlation between bile and tissue methylation levels. 9](#_Toc222754272)

[Fig. S6 Box plot – normalized DNA methylation levels. 10](#_Toc222754273)

[Fig. S7 ROC curves - bile samples from sporadic CCA. 10](#_Toc222754274)

[Fig. S8 Box plot – TCGA data. 11](#_Toc222754275)

[Fig. S9 Droplet Digital PCR plot. 12](#_Toc222754276)

[**References** 13](#_Toc222754277)

#

# **Supplementary Methods**

## ***DNA isolation***

*Tissue and bile:* DNA was extracted from ~25 mg of fresh frozen tissue using the AllPrep DNA/RNA kit (Qiagen, Hilden, Germany), following the manufacturers recommendations. DNA concentrations were measured on a ND-1000 Nanodrop Spectrophotometer (NanoDrop Technologies, Wilmington, DE). For bile, DNA was extracted from 100-200 µl of bile using a standard Phenol chloroform procedure. Briefly, bile was lysed using EDTA, nucleic lysis solution, RNase A, and Proteinase K. The lysate was extracted twice with phenol:chloroform:isoamyl alcohol (25:24:1) using Phase Lock Gel tubes (5prime PLG tube, Quantabio, Beverly, Massachusetts), with centrifugation at 16,000 g for 5 min at room temperature. This was followed by two extractions with chloroform. The final aqueous phase was precipitated with glycogen, ammonium acetate, and cold ethanol, then incubated at -20 °C for at least 2 h. After centrifugation, the DNA pellet was washed with 70% ethanol, dried at 37 °C, and resuspended in 35–70 µL TE buffer (pH 8.0). DNA purity was assessed by NanoDrop, and DNA concentrations were measured using the Qubit dsDNA high sensitivity assay or the Qubit dsDNA broad range assay (Thermo Fisher Scientific). The median DNA yield was 2022 ng per sample (range: 78 ng – 171 µg). DNA from both tissue and bile were stored at 4 ℃.

*Plasma*: cell-free DNA (cfDNA) was extracted from 2 mL plasma using QIAamp Circulating Nucleic Acid Kit (Qiagen) according to the manufacturer’s protocol. A QIAcube Connect (Qiagen) was used for automated clean-up. The cfDNA was first eluted in 150 µL AVE-buffer, and subsequently concentrated to a final volume of 20–40 µL using Vivacon 500 centrifugal filters (30,000 MWCO; Sartorius, Göttingen, Germany) by centrifugation at 5,000 g for 6 minutes. DNA concentrations were measured on a Qubit Fluorometer using the Qubit (1X) dsDNA High Sensitive Assay (ThermoFisher Scientific). The size distribution of the isolated DNA was assessed using an Agilent 2200 TapeStation system, with the D1000 ScreenTape and the Cell-free DNA ScreenTape Assays (Agilent). The median DNA yield was 42.5ng per sample (range: 6.9 ng - 434 ng). The isolated plasma cfDNA was stored at -20 ℃.

## ***Reduced representation bisulfite sequencing***

*Pre-processing:* Demultiplexed data in the form of FASTQ files, prepared using bcl2fastq (RRID: SCR_015058), was imported to TSD (Tjenester/sevices for Sensitive Data, University of Oslo). Quality control of the raw sequencing data was done using FastQC 0.11.2 (<https://www.bioinformatics.babraham.ac.uk/projects/fastqc/>).

Adaptors and low-quality reads were trimmed using Trim Galore! 0.6.1 (<https://github.com/FelixKrueger/TrimGalore>), and pseudorandom bases added between the sequencing primer and the inserts were subsequently trimmed using the script “trimRRBSdiversityAdaptCustomers.py” provided by NuGEN (Tecan). FastQC was used for quality control of each trimming step. Trimmed reads were aligned to a bisulfite converted version of the human genome GRCh38 using Bismark 0.17.0 ^[1]^. After alignment the resulting Sequence Alignment Map (SAM) files from each sequencing run was merged into one SAM-file per sample, and sorted using Samtools 1.3.1 ^[2]^. The script “strip_bismark_sam.sh” (NuGEN, Tecan) was used to prepare the SAM-files for removal of PCR duplicates, which in turn was performed using the tool NuDup provided by NuGEN (Tecan).

*Selection of candidate biomarkers from RRBS data:* DMRfinder initially identified 11632 differentially methylated regions. To prioritize biomarker candidates, two complimentary filtering strategies were applied. The first strategy selected regions showing minimal DNA methylation in PSC but detectable DNA methylation in CCA. Specifically, at least eight of the nine PSC samples were required to have a methylation ratio below 5% for the region in question, while at least nine of ten CCA samples had to exceed a ratio of 5%. This approach yielded 27 candidate regions. The second strategy identified candidate regions with maximal methylation difference between CCA and PSC by retaining only regions where all CCA samples had higher methylation values than any PSC sample, and where the CCA samples showed at least 5-fold higher mean methylation level than the PSC samples. Applying this strategy provided 13 candidates. In parallel MethylKit identified 4371 windows of 1000 bp and 2375 windows of 200 bp that were significantly more methylated in CCA relative to PSC. Regions were selected according to the same two conceptual strategies described above. The first strategy yielded 27 candidates from the 1000‑bp windows (5 non-overlapping) and 12 from the 200‑bp windows (8 non-overlapping), while the second provided 51 candidates from the 1000-bp approach (13 non-overlapping) and 18 candidate regions from the 200‑bp approach (14 non-overlapping). Overlapping regions among the two window sizes and between DMRfinder and methylKit results were merged to create a non‑redundant biomarker candidate list (n=61). These regions were visually inspected using the Integrative Genomics Viewer (IGV) to confirm consistent CpG‑level methylation differences between CCA and PSC and to ensure suitability for downstream assay design. Based on this inspection, 12 ddPCR assays were designed, targeting four regions identified by DMRfinder (three from strategy 1 and one from strategy 2), and eight regions identified from methylKit (four from strategy 1 and four from strategy 2).

## ***Droplet digital PCR***

*Droplet generation:* Droplet generation of 20 µL reaction mixture was performed using an Automated Droplet Generator (BioRad). A PX1 PCR Plate Sealer was used for sealing of 96-well PCR plates (BioRad). The PCR was performed either in a T100 Thermal Cycler or in a PTC Tempo Thermal Cycler, with thermal cycling conditions as described in Supplementary Table 17. Droplets were read using the QX200 Droplet Reader (tissue and bile) or the QX600 Droplet Reader (plasma). All reagents utilized were sourced from BioRad.

*Controls and calculation of methylation concentrations:* For all analyses, methylation-positive controls (universal methylated human DNA standard; Zymo Research, Irvine, CA, USA), methylation-negative controls (human WGA non-methylated DNA; Zymo Research), and non-template controls (NTC; RNase-free water; Sigma Aldrich, St. Louis, MO, USA) were included. Positive droplets were called using the PoDCall algorithm (<https://bioconductor.org/packages/PoDCall/>). The methylation concentrations (copies per µL) were normalized by dividing the concentration of the target (*i.e*., the biomarker) by the concentration of the 4Plex internal control and multiplying by a constant of 400 as previously described ^[3]^. For tissue and bile, samples with <3 positive droplets for the target gene were censored and the normalized DNA methylation value was set to 0, while for plasma a threshold of <2 positive droplets was used for censoring. From bile samples, four patients displayed missing values for one biomarker; 3 PSC alone and 1 disease control. These patients were excluded from analyses for the specific marker. Examples of ddPCR plot, including positive and negative experimental results, can be found in Supplementary Figure 5.

# **Supplementary Discussion**

*Biomarkers: PRKCB,* a member of the protein kinase C family, regulates cell growth and apoptosis. It has previously been shown to be hypermethylated in CCA and other cancers, and has been linked to immune status and immunotherapy response ^[4, 5]^. *KCNA6* encodes a potassium channel. Altered potassium channel expression has been linked to cancer cell proliferation and migration ^[6]^. *KCNA6* hypermethylation has been found in pancreatic ^[7]^ and esophageal adenocarcinomas ^[8]^. Finally, zinc finger proteins, including *ZFP82*, play key roles in transcriptional regulation and tumor suppression. *ZFP82* has been identified as a functional tumor suppressor disrupted by promoter hypermethylation in gastric- ^[9]^, esophageal- ^[10]^, and pancreatic cancers ^[11]^.

# **Supplementary Figures**

**
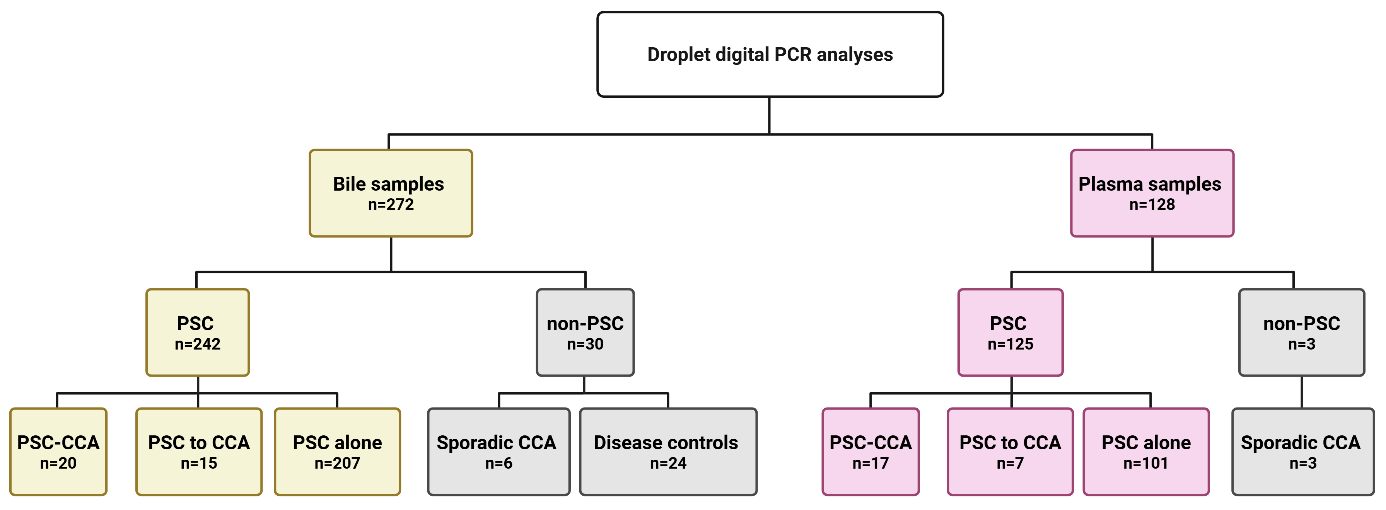
**

**Fig. S1 Flowchart.** Flowchart of bile (n=272) and plasma (n=128) samples included in the study and analysed with droplet digital PCR for DNA methylation biomarkers. One bile and one plasma sample were included per patient. Bile and plasma samples are from the same patient cohort, but not necessarily taken at the same time point. Abbreviations: PSC, primary sclerosing cholangitis; CCA, cholangiocarcinoma; PSC-CCA, bile sampling performed <3 months before diagnosis of CCA; PSC to CCA, bile sampling performed 3-36 months before a diagnosis of CCA; PSC alone, no signs of CCA observed >36 months after bile sampling; disease controls - other liver diseases including hereditary, idiopathic non-alcoholic fatty liver, biliary stone and autoimmune liver disease other than PSC. Created in BioRender. Vedeld, H. M. (2026) https://BioRender.com/vq16osc


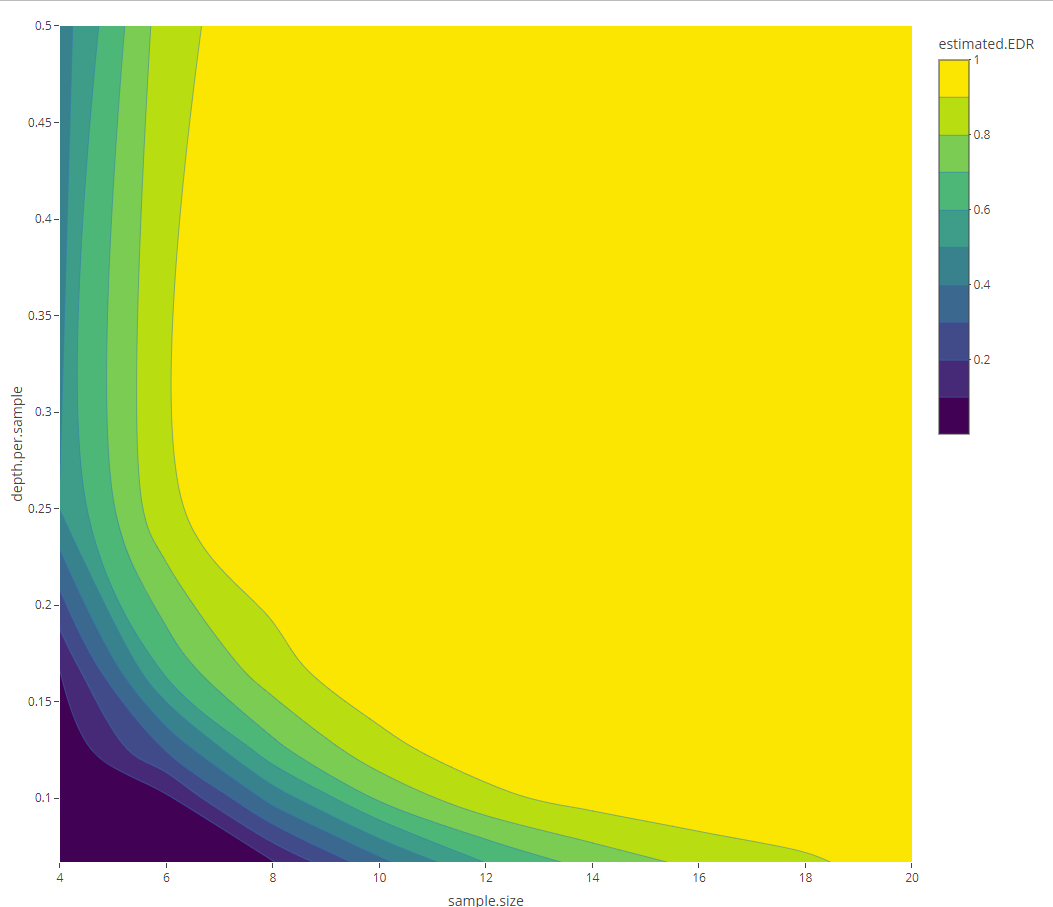


**Fig. S2 Contour plot for genome wide power.** Contour plot of genome wide power for detecting differentially methylated regions using simulated data, as estimated with the R package MethylSeqDesign. The x axis shows the number of samples per group (controls or cases). The y axis shows the sequencing depth at a given samples size per group, expressed as a fraction of one full sequencing lane, with larger values corresponding to deeper sequencing. The color scale indicates the expected discovery rate (EDR), i.e. the proportion of truly differentially methylated regions that are expected to be detected at a false discovery rate threshold of 0.05. Abbreviations: EDR, expected discovery rate (power).


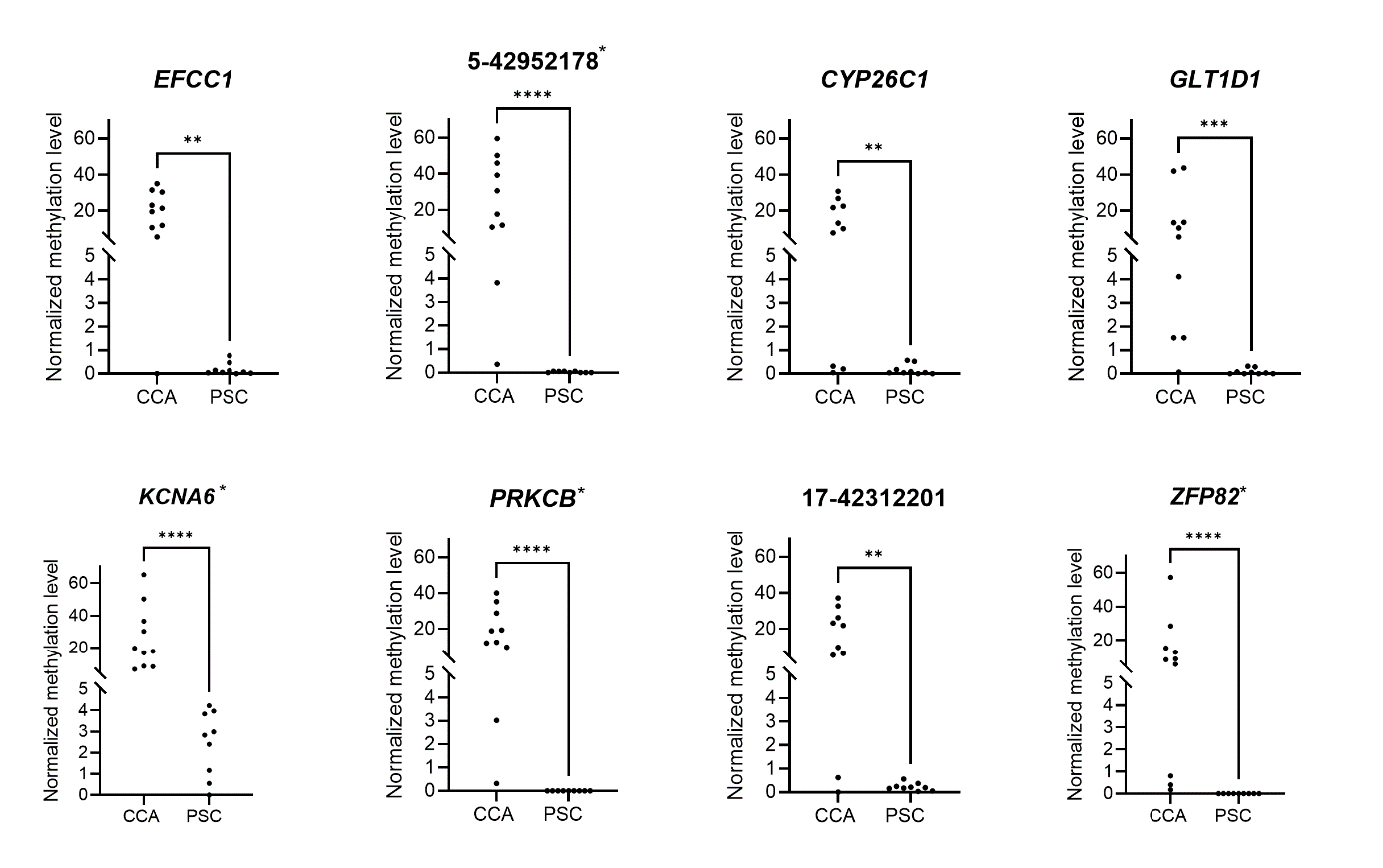


**Fig. S3 Tree plot of normalized DNA methylation levels in tissue.** Normalized DNA methylation levels for eight assays in 10 cholangiocarcinoma (CCA) and 9 primary sclerosing cholangitis (PSC) tissue samples. * Denotes assays which were included in analyses of bile and plasma samples. Levels of significance: **, p<0.005; ***, p<0.001; ****, p<0.0001 (Mann-Whitney test). Abbreviations: CCA, cholangiocarcinoma; PSC, primary sclerosing cholangitis.


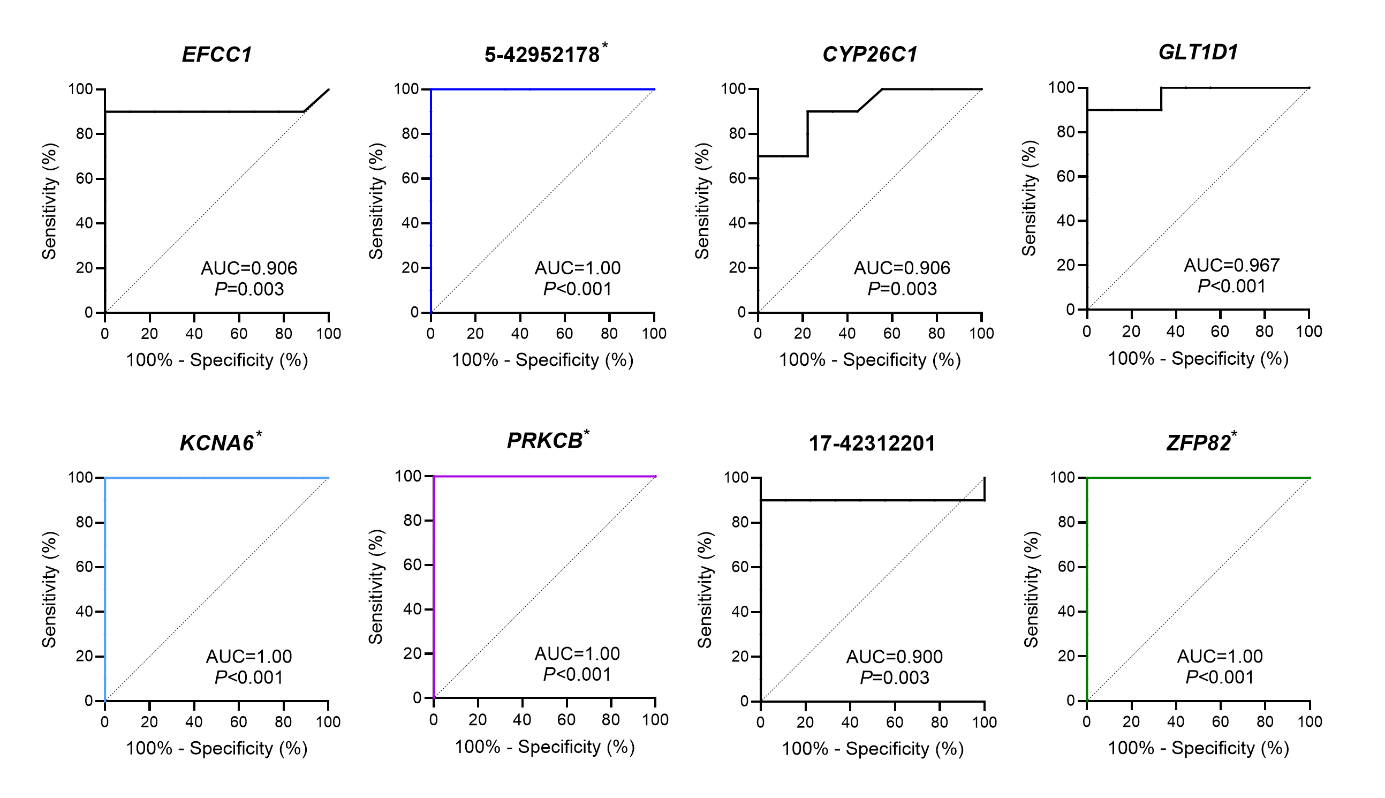


**Fig. S4 ROC curves - tissue samples.** ROC curves and AUCs for eight DNA methylation assays tested in 10 cholangiocarcinoma (CCA) and 9 primary sclerosing cholangitis (PSC) tissue samples, generated in GraphPad. The normalized DNA methylation values for CCA samples are plotted against the normalized DNA methylation values for PSC samples. *Denotes assays which were included in analyses of bile and plasma samples. Abbreviations: AUC, area under the ROC curve.


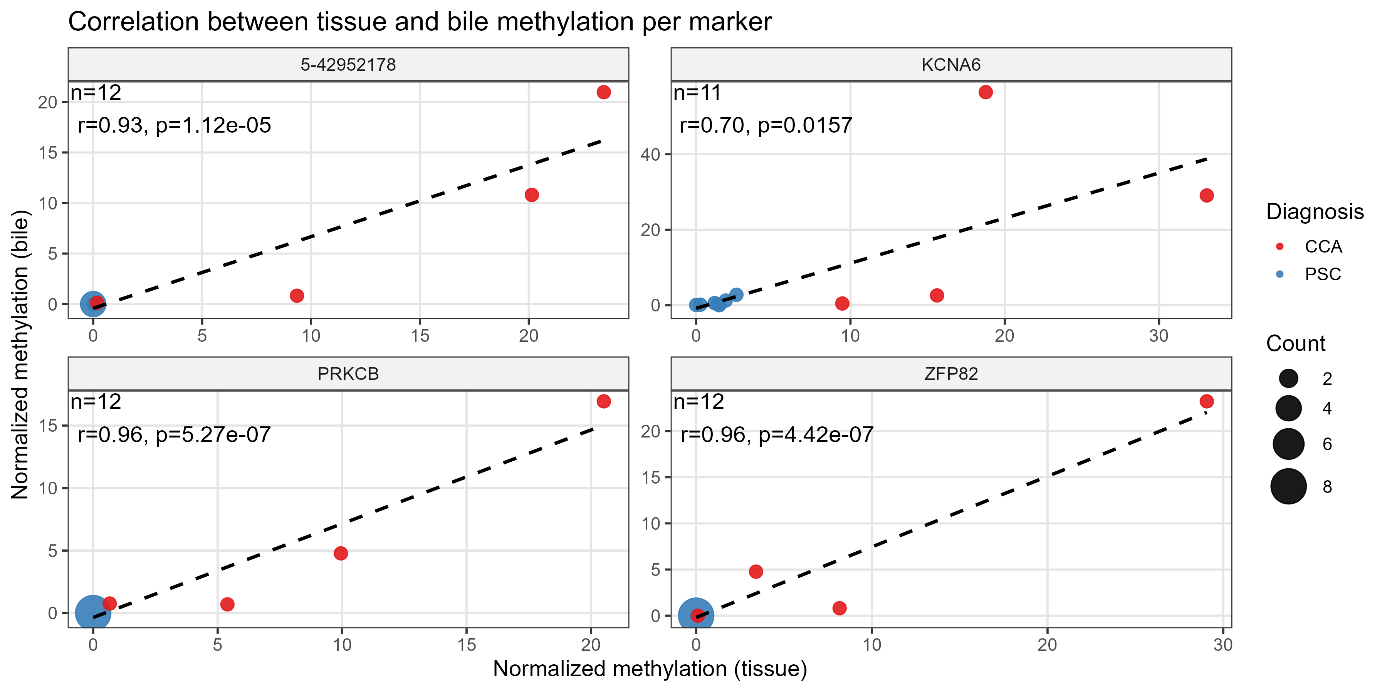


**Fig. S5 Correlation between bile and tissue methylation levels.** Correlation between normalized DNA methylation levels in tumor tissue and matched bile for the four biomakers 5-42952178, *KCNA6*, *PRKCB*, and *ZFP82*. Each point represents one individual; color indicates diagnosis (PSC or CCA). A linear regression line (dashed) is shown per panel and Pearson correlation coefficient (r), p-value and sample size (n) are indicated in each panel. Pearson correlations were computed with cor.test. Note that bile and tissue samples were not always collected at the same time point; the time interval between sampling ranged from 0.5 to 10.1 months for the CCA cases (median = 1.7 months) and from 0 to 20.1 months for the PSC cases (median = 3.4 months).

**
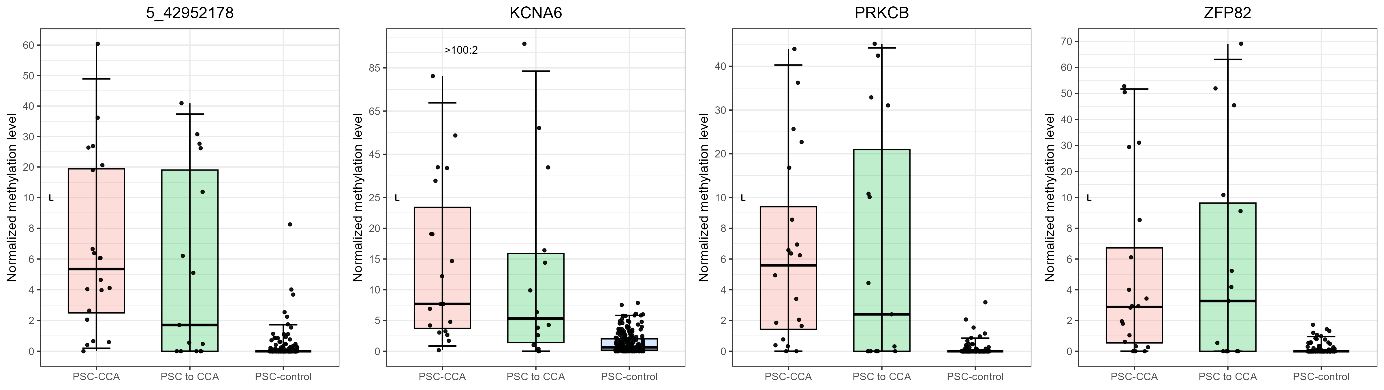
** **Fig. S6 Box plot – normalized DNA methylation levels.** Normalized DNA methylation levels for the four biomakers 5-42952178, *KCNA6*, *PRKCB*, and *ZFP82* in primary sclerosing cholangitis (PSC) and cholangiocarcinoma (CCA). The median methylation value is shown as a horizontal bar and 95% confidence intervals for the median are indicated by vertical error bars. Values >100 are excluded. The y-axis is broken and stretched in the lower range to better show variation among low values (indicated by “L”), while the upper range is compressed. Abbreviations: PSC-CCA, PSC with concomitant CCA; PSC to CCA, PSC clinically diagnosed with CCA within 36 months after bile sampling; PSC alone, PSC without any malignancy.


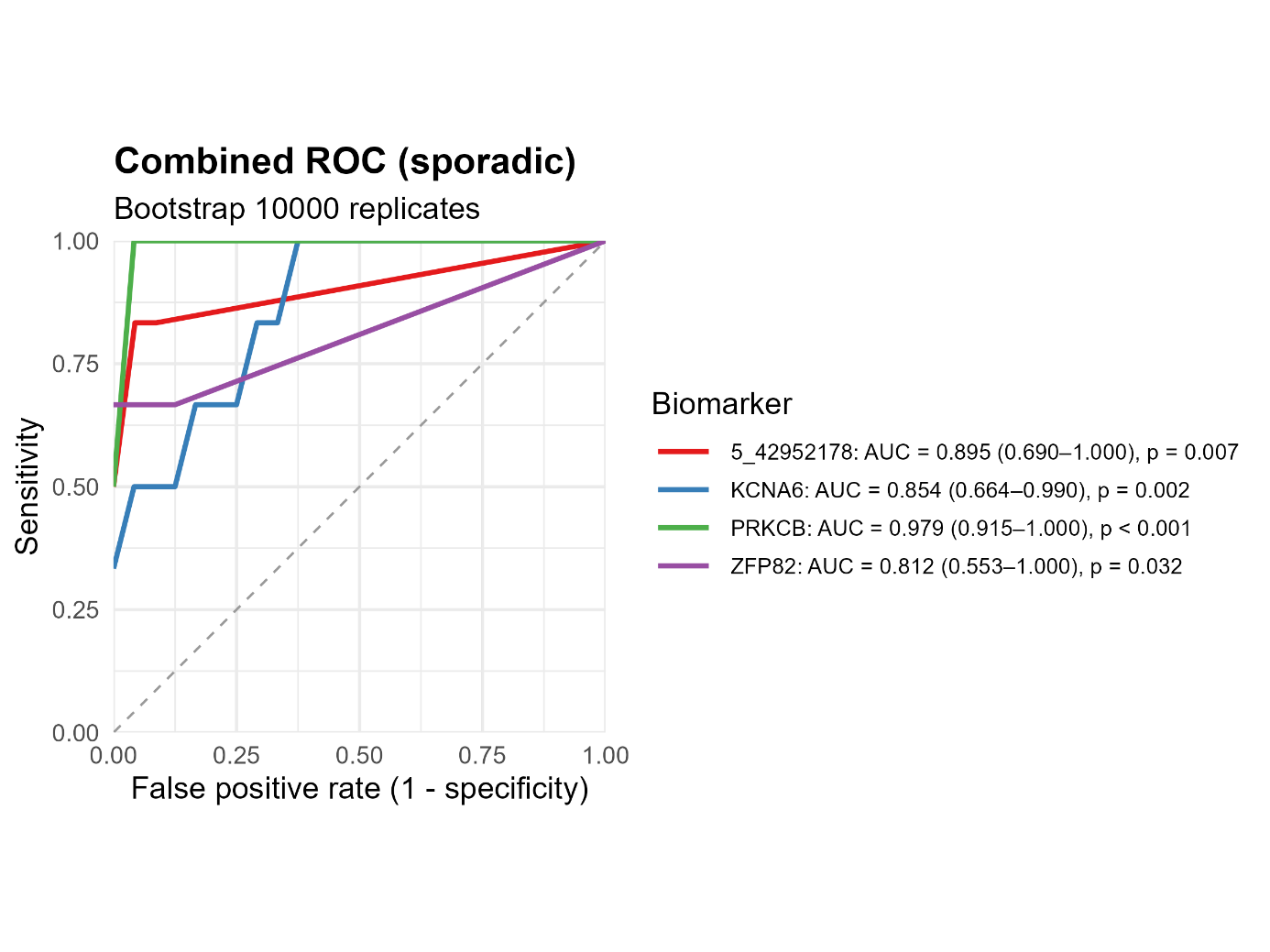
 **Fig. S7 ROC curves - bile samples from sporadic CCA.** Combined ROC curves for biomarkers analyzed in bile from individuals with sporadic cholangiocarcinoma (CCA; n=6) and disease controls (n=24). ROC curves and AUCs were computed in R (pROC package). Ninety-five percent confidence intervals (CIs) were estimated by bootstrapping (B=10,000; boot package). Two-sided bootstrap p-values were calculated for the null hypothesis AUC=0.5, and p <0.05 was considered statistically significant. Abbreviations: AUC, area under the ROC curve.


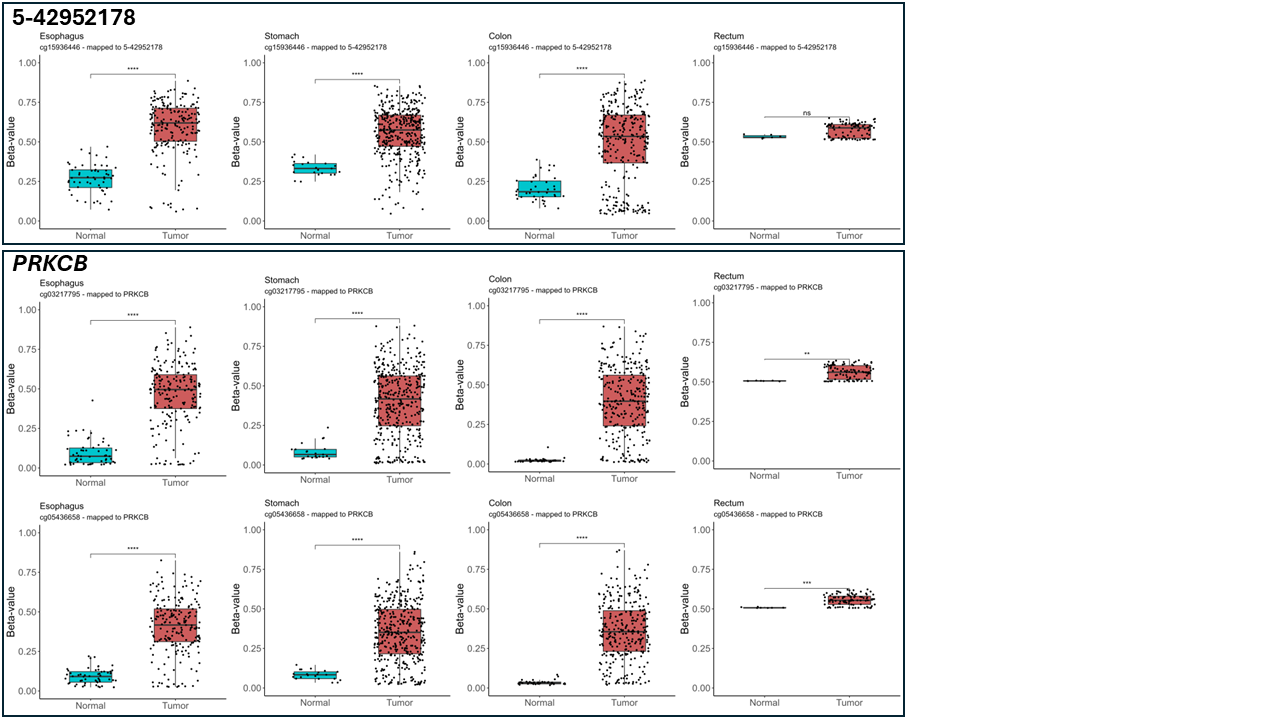


**Fig. S8 Box plot – TCGA data**. Distribution of DNA methylation levels in normal and tumor samples from four gastrointestinal adenocarcinomas in The Cancer Genome Atlas (TCGA; obtained from the NCI Genomic Data Commons (<https://gdc.cancer.gov/>), granted through dbGaP to HPB as a collaborator in the study (phs000178.v11.p8)). Probes on the Illumina HumanMethylation450 BeadChip array annotated to 5-42952178 (cg15936446) and *PRKCB* (cg03217795 and cg05436658) according to the Illumina manifest are shown. No probes are annotated to *KCNA6* or *ZFP82* on this array. Level of significance: **p <0.01; ***p <0.001; ****p <0.0001; ns, not significant (Wilcoxon rank‑sum test).


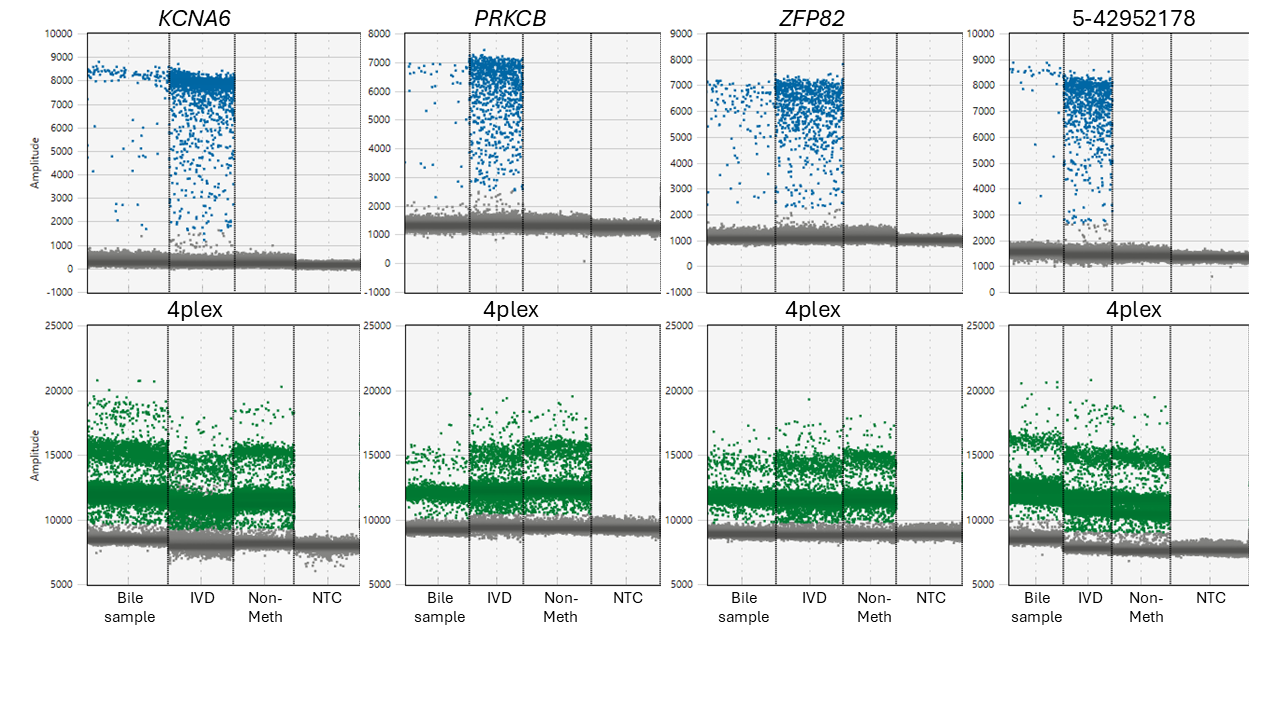


**Fig. S9 Droplet Digital PCR plot.** Example of droplet digital PCR plots, including negative and positive experimental results. Positive droplets were called using the PoDCall algorithm (https://bioconductor.org/packages/PoDCall/).

Abbreviations: IVD, universal methylated human DNA standard; non-meth, human WGA non-methylated DNA; NTC, non-template control

# **References**

[1] Krueger F, Andrews SR. Bismark: a flexible aligner and methylation caller for Bisulfite-Seq applications. Bioinformatics (Oxford, England). 2011;27:1571–1572.

[2] Li H, Handsaker B, Wysoker A, et al. The Sequence Alignment/Map format and SAMtools. Bioinformatics (Oxford, England). 2009;25:2078–2079.

[3] Pharo HD, Andresen K, Berg KCG, et al. A robust internal control for high-precision DNA methylation analyses by droplet digital PCR. Clin Epigenetics. 2018;10:24.

[4] Abdelatty A, Sun Q, Hu J, et al. Pan-Cancer Study on Protein Kinase C Family as a Potential Biomarker for the Tumors Immune Landscape and the Response to Immunotherapy. Front Cell Dev Biol. 2021;9:798319.

[5] Yang JD, Ghoz H, Aboelsoud MM, et al. DNA Methylation Markers for Detection of Cholangiocarcinoma: Discovery, Validation, and Clinical Testing in Biliary Brushings and Plasma. Hepatology communications. 2021;5:1448–1459.

[6] Dupuy M, Gueguinou M, Potier-Cartereau M, et al. SK(C)(a)- and Kv1-type potassium channels and cancer: Promising therapeutic targets? Biochem Pharmacol. 2023;216:115774.

[7] Bararia A, Maiti A, Ghosh G, et al. Identification of KCNJ5 gene an adverse prognosis associated novel onco-ionchannel in Indian pancreatic cancer cohort. Discov Oncol. 2025;16:236.

[8] Ouadid-Ahidouch H, Rodat-Despoix L, Matifat F, et al. DNA methylation of channel-related genes in cancers. Biochimica et biophysica acta. 2015;1848:2621–2628.

[9] Wang S, Cheng Y, Du W, et al. Zinc-finger protein 545 is a novel tumour suppressor that acts by inhibiting ribosomal RNA transcription in gastric cancer. Gut. 2013;62:833–841.

[10] Ye L, Xiang T, Fan Y, et al. The 19q13 KRAB Zinc-finger protein ZFP82 suppresses the growth and invasion of esophageal carcinoma cells through inhibiting NF-κB transcription and inducing apoptosis. Epigenomics. 2019;11:65–80.

[11] Sun H, Xin R, Zheng C, et al. Aberrantly DNA Methylated-Differentially Expressed Genes in Pancreatic Cancer Through an Integrated Bioinformatics Approach. Frontiers in genetics. 2021;12:583568.
