# Supplementary material for: Accurate detection of cholangiocarcinoma in primary sclerosing cholangitis using DNA methylation biomarkers in bile and plasma
Source: JHEP Rep. 2026 Apr 29;8(8):101876. doi: 10.1016/j.jhepr.2026.101876 (PMC13380102; doi:10.1016/j.jhepr.2026.101876)
Supplement: Multimedia component 2 [file mmc2.docx]

**JHEP Reports**

**CTAT methods**

Tables for a “Complete, Transparent, Accurate and Timely account” (CTAT) are now mandatory for all revised submissions. The aim is to enhance the reproducibility of methods.

- Only include the parts relevant to your study
- Refer to the CTAT in the main text as ‘Supplementary CTAT Table’
- Do not add subheadings
- Add as many rows as needed to include all information
- Only include one item per row

**If the CTAT form is not relevant to your study, please outline the reasons why:**

| All information found in this form is also available in the manuscript |
| --- |

- 1. **Antibodies**

| **Name** | **Citation** | **Supplier** | **Cat no.** | **Clone no.** |
| --- | --- | --- | --- | --- |
|  |  |  |  |  |

- 1. **Cell lines**

| **Name** | **Citation** | **Supplier** | **Cat no.** | **Passage no.** | **Authentication test method** |
| --- | --- | --- | --- | --- | --- |
|  |  |  |  |  |  |

- 1. **Organisms**

| **Name** | **Citation** | **Supplier** | **Strain** | **Sex** | **Age** | **Overall n number** |
| --- | --- | --- | --- | --- | --- | --- |
|  |  |  |  |  |  |  |

- 1. **Sequence based reagents**

| **Name** | **Sequence** | **Supplier** |
| --- | --- | --- |
|  |  |  |

- 1. **Biological samples**

| **Description** | **Source** | **Identifier** |
| --- | --- | --- |
| **Tissue** | Oslo University Hospital (OUS), Rikshospitalet, Oslo, Norway | Pseudonymised patient identifier – supplementary Table 1 |
| **Bile** | Oslo University Hospital (OUS), Rikshospitalet, Oslo, Norway; Karolinska University Hospital, Stockholm, Sweden; Helsinki University Hospital, Helsinki, Finland | Pseudonymised patient identifier – supplementary Table 2 |
| **Plasma** | Oslo University Hospital (OUS), Rikshospitalet, Oslo, Norway | Pseudonymised patient identifier – supplementary Table 4 |

- 1. **Deposited data**

| **Name of repository** | **Identifier** | **Link** |
| --- | --- | --- |
| Zenodo |  | Will be made available upon article acceptance |

- 1. **Software**

| **Software name** | **Manufacturer** | **Version** |
| --- | --- | --- |
| R | R Project | 4.0.0/ 4.4.2 |
| GraphPad Prism | GraphPad Software | 10.4.2 |
| PodCall algorithm | Bioconductor | 1.15.0 /1.15.2 |
| bcl2fastq | Illumina | 2.20 |
| FastQC | Babraham Institute | 0.11.2 |
| Trim Galore! | Felix Krueger | 0.6.1 |
| Bismark | Babraham Institute | 0.17.0 |
| Samtools | Genome Research Ltd (Wellcome Sanger Institute) | 1.3.1 |
| NuDup | NuGEN (Tecan) | NA |
| methylKit | Altuna Akalin, Weill Cornell Medical College | 1.14.x |
| DMRfinder | Gaspar JM, Hart RP | 0.3 |

- 1. **Other (*e.g*. drugs, proteins, vectors etc.)**

|  |  |  |
| --- | --- | --- |
|  |  |  |

- 1. **Please provide the details of the corresponding methods author for the manuscript:**

| Guro E Lind, Department of Molecular Oncology, Institute for Cancer Research, Oslo University Hospital - Norwegian Radium Hospital, Montebello, 0379 Oslo, Norway. Telephone: +47 22781729, Fax: +47 22935767, E-mail: g.e.lind@ibv.uio.no |
| --- |

**2.0 Please confirm for randomised controlled trials all versions of the clinical protocol are included in the submission. These will be published online as supplementary information.**

|  |
| --- |
